# The Role of Lipid Disorders in Cardiovascular Mortality Among Saudis: A Review Perspective

**DOI:** 10.1155/ije/5596073

**Published:** 2026-06-24

**Authors:** Aseel Awad Alsaidan

**Affiliations:** ^1^ Department of Family and Community Medicine, College of Medicine, Jouf University, Sakaka, 72388, Aljouf, Saudi Arabia, ju.edu.sa

**Keywords:** cardiovascular, lipid, mortality, Saudi Arabia

## Abstract

Cardiovascular diseases (CVD) are the main cause of death worldwide and in Saudi Arabia, accounting for about 31% of all deaths globally and 45% of deaths in the Kingdom. The aim of this review was to review the epidemiology, pathophysiology, and management of lipid disorders (mainly dyslipidemia) which are major contributors to CVD in Saudi Arabia. The unique risk profile of the Saudi population with a high prevalence of dyslipidemia (especially low high‐density lipoprotein cholesterol [HDL‐C] and high triglycerides), in addition to the genetic factors, urbanization, sedentary lifestyle, and change in diet to a western style has been described. National surveys showed prevalence rates of dyslipidemia > 50% in adults and high prevalence in adolescents with high regional and gender differences. Low HDL‐C was the most common and important lipid abnormality, which was significantly related to all‐cause and CVD mortality and independently predicts adverse outcomes. This review summarizes the limits of the current treatment strategy that mainly focuses on LDL‐C lowering and shows the need for the development of new therapies targeting HDL‐C and triglycerides. The public health implications include an increasing economic burden, the need for early screening, culture‐adapted prevention programs, and integration of genetic risk prediction. Large‐scale epidemiological studies, national registries, and personalized medicine approaches are needed to fill the gaps in knowledge and optimize management. Implementing population‐based programs is a must to decrease the burden of CVD and improve the outcome in Saudi Arabia which may be an example for other countries experiencing the same epidemiological transition.

## 1. Introduction

Cardiovascular diseases (CVD) remain the leading cause of mortality worldwide, accounting for approximately 17.9 million deaths annually and nearly 31% of all global deaths [1]. In Saudi Arabia, the burden of CVD is particularly pronounced, with cardiovascular conditions responsible for approximately 45% of total mortality, reflecting a growing public health challenge associated with rapid socioeconomic development, urbanization, and lifestyle transitions [2, 3]. These shifts have contributed to a marked increase in cardiometabolic risk factors, including obesity, diabetes mellitus, hypertension, and dyslipidemia, all of which are strongly associated with adverse cardiovascular outcomes.

Dyslipidemia, defined by abnormalities in plasma lipoproteins such as elevated low‐density lipoprotein cholesterol (LDL‐C), elevated triglycerides (TG), and reduced high‐density lipoprotein cholesterol (HDL‐C), represents a major modifiable risk factor for atherosclerotic CVD (ASCVD) [4]. Extensive epidemiological evidence indicates that dyslipidemia is highly prevalent in Saudi Arabia, affecting more than half of the adult population, with distinct patterns characterized by low HDL‐C levels and hypertriglyceridemia [5–8]. These lipid abnormalities are closely linked to the high prevalence of obesity, physical inactivity, and dietary patterns increasingly dominated by refined carbohydrates and saturated fats.

Saudi Arabia presents a unique epidemiological and genetic context for the study of lipid disorders and cardiovascular risk, combined with lifestyle changes characterized by reduced physical activity and adoption of Western diets, which is a diet characterized by the heavy consumption of refined sugars, refined vegetable oils, dairy, and processed cereals. These modern staples have fundamentally altered key nutritional profiles—such as glycemic load and fatty acid composition—leading to an evolutionary discordance that promotes chronic diseases. Population‐based studies demonstrate substantial regional, age‐related, and gender‐specific variations in lipid profiles, suggesting the influence of both environmental and biological determinants [7–9]. Moreover, genetic predisposition plays an important role in shaping cardiovascular risk in the Saudi population. Genome‐wide and candidate‐gene studies have identified several genetic variants associated with dyslipidemia, hypertension, and premature CVD, findings further supported by national initiatives such as the Saudi Mendelian Genomics Project [10]. High rates of consanguinity may amplify the expression of inherited lipid disorders, underscoring the need for population‐specific risk assessment strategies [11].

The pathophysiological link between dyslipidemia and CVD is primarily mediated through the development and progression of atherosclerosis. Lipid abnormalities promote endothelial dysfunction, lipid retention within the arterial wall, chronic vascular inflammation, and plaque formation, processes that collectively increase the risk of coronary artery disease (CAD), stroke, and heart failure [4, 12]. Importantly, while LDL‐C has traditionally been the primary therapeutic target, growing evidence suggests that residual cardiovascular risk persists in many patients despite optimal LDL‐C lowering, particularly in populations with high TG levels and low HDL‐C, such as those observed in Saudi Arabia. Clinical evidence also strongly suggests that cardiovascular events are significantly reduced through the lowering of LDL‐C and TG levels; however, the impact of isolated HDL‐C elevation on cardiovascular outcomes remains less certain [4, 5, 12].

Despite the availability of effective lipid‐lowering therapies, including statins and adjunctive pharmacological agents, the burden of dyslipidemia‐related cardiovascular morbidity and mortality remains high in Saudi Arabia. This highlights limitations in current prevention and management strategies and emphasizes the need for comprehensive approaches that integrate epidemiological evidence, pathophysiological insights, genetic risk profiling, and culturally adapted lifestyle interventions. Furthermore, gaps persist in long‐term longitudinal data, national registries, and Saudi‐specific cardiovascular risk prediction tools, limiting the ability to optimize prevention and treatment strategies.

The aim of this review is to synthesize current evidence on the epidemiology, pathophysiology, and clinical impact of lipid disorders in Saudi Arabia, with a particular focus on their association with cardiovascular mortality. By examining population‐specific lipid patterns, underlying mechanisms, and existing management approaches, this review seeks to identify knowledge gaps and highlight future research directions. Understanding the unique lipid profile and cardiovascular risk landscape of the Saudi population may inform more effective prevention strategies and provide insights applicable to other countries undergoing similar epidemiological transitions.

## 2. Epidemiology of Lipid Disorders in Saudi Arabia: Prevalence, Global Comparisons, and Lifestyle Impacts

Over the past several decades, Saudi Arabia has undergone a marked epidemiological transition characterized by a rising burden of noncommunicable diseases, including dyslipidemia. Lipid abnormalities, such as elevated LDL‐C, low HDL‐C, hypertriglyceridemia, and increased total cholesterol (TC), are highly prevalent and are strongly associated with CVD risk rather than acting as direct causal determinants. This section synthesizes contemporary evidence on the epidemiology of lipid disorders in Saudi Arabia, compares national patterns with regional and global trends, and evaluates the impact of lifestyle‐related factors on lipid profiles [6–8].

### 2.1. Prevalence of Lipid Disorders in Saudi Arabia

#### 2.1.1. Adult Population

Dyslipidemia is defined in this review according to the 2019 ESC/EAS guidelines. Under these criteria, individuals are classified as dyslipidemic if they present with one or more of the following: TC > 5.0 mmol/L, LDL‐C > 3.0 mmol/L, and TG > 1.7 mmol/L. Furthermore, low HDL‐C is defined as < 1.0 mmol/L for males and < 1.2 mmol/L for females.

Dyslipidemia is highly prevalent among Saudi adults. A national cross‐sectional survey involving 4490 participants reported that 44% of adults had hypertriglyceridemia (≥ 1.7 mmol/L), while 20% had elevated TC (≥ 5.2 mmol/L) [6]. Pronounced gender differences have been consistently observed, with hypertriglyceridemia affecting 47.6% of males compared to 33.7% of females, and low HDL‐C present in 44.7% of males versus 33.2% of females. The prevalence of hypercholesterolemia (54.9%) and hypertriglyceridemia (47.6%) reaches its peak in the Northern and Eastern regions, which contrasts with the lower rates observed in the Southern region. According to the Saudi Census 2022 (GASTAT, 2023), these regional disparities correlate with population density and urbanization patterns. The Eastern region is one of the Kingdom’s primary urban and industrial hubs, housing a large portion of the 82% of Saudis now living in urban centers. Conversely, the Southern region maintains a lower population density and a more traditional, rural landscape. This suggests that the higher lipid abnormalities in the North and East are driven by the ‘urbanized’ lifestyle—characterized by sedentary occupations and a higher intake of processed foods—that is less prevalent in the rural South [4].

#### 2.1.2. Adolescent and Pediatric Populations

Lipid abnormalities are detectable early in life among the Saudi population. A national study of 5854 adolescents aged 10–19 years found an overall dyslipidemia prevalence of 25.5%, with higher rates in males (33.3%) than females (17.9%) [9]. The most frequently reported abnormalities included hypertriglyceridemia (9.6%) and low HDL‐C (12.8%), while elevated non‐HDL‐C was observed in 8.3% of participants, highlighting early exposure to atherogenic lipid profiles. Geographic variations in lipid profiles are starkly evident; for instance, the dyslipidemia prevalence rate stands at 44.5% in Najran, whereas Al‐Bahah reports a significantly lower rate of 14.1%. This disparity can be better understood by examining the demographic profiles provided by the Saudi Census 2022 (GASTAT, 2023). Najran, with a population of approximately 592,000, has undergone rapid urban expansion. In contrast, Al‐Bahah, though having a population of roughly 435,000, remains one of the more rural and mountainous regions of the Kingdom. The lower prevalence in Al‐Bahah may reflect a more traditional lifestyle and higher levels of physical activity associated with its geographic terrain, compared to the more urbanized environment of Najran [13].

#### 2.1.3. Special Populations

A significant overlap exists between metabolic disorders and lipid abnormalities, which substantially elevates vascular risk. For instance, over 70% of individuals with Type 2 diabetes mellitus (T2DM) present with co‐occurring dyslipidemia [14]. Similarly, a Riyadh‐based study identified hyperlipidemia in 40.7% of newly diagnosed psoriatic arthritis patients. These associations suggest that chronic inflammatory states and metabolic dysregulation are closely linked to lipid processing issues in the Saudi population, collectively compounding the risk of cardiovascular events [15].

### 2.2. Global and Regional Comparisons

#### 2.2.1. Middle East and North Africa (MENA) Context

Dyslipidemia has reached epidemic levels in the MENA region, and current data shows a pooled prevalence rate of 54.08% (95% CI: 43.83–66.71) [16]. The most prevalent lipid abnormalities are hypertriglyceridemia (32.51%) and low HDL‐C (44.71%), reflecting shared dietary patterns and sedentary lifestyles. Saudi Arabia demonstrates dyslipidemia rates comparable to regional averages but higher than those reported in some neighboring Gulf countries such as Kuwait and Oman [17]. Comparative prevalence data for Saudi Arabia, the MENA region, and global estimates are summarized in Table 1.

**TABLE 1:** Comparative prevalence of lipid abnormalities.

Parameter	Saudi Arabia	MENA region	Global average
Dyslipidemia	54.0% [7]	54.08% [16]	39% [17]
Hypertriglyceridemia	44% [6]	32.51% [16]	25% [17]
Low HDL‐C	44.7% [7]	44.71% [16]	30% [17]
Elevated LDL‐C	32.1% [7]	32.09% [16]	28% [17]

#### 2.2.2. Temporal Trends

Since 1990, age‐standardized mortality attributable to high LDL‐C has declined by 26.5%, and mortality related to elevated systolic blood pressure has decreased by 23.4%. In contrast, metabolic risk factors such as obesity and hyperglycemia have increased by 5.1% and 21.4%, respectively, indicating a shifting cardiometabolic risk profile [17]. This pattern underscores a dual burden in Saudi Arabia, where traditional lipid‐related risks persist alongside emerging metabolic threats.

### 2.3. Impact of Lifestyle Factors on Lipid Profiles

#### 2.3.1. Dietary Habits

The transition toward Westernized dietary patterns has been associated with adverse lipid profiles. More than half of the Saudi population consumes carbonated beverages daily, while fruit and vegetable intake remains below recommended levels [18, 19]. High consumption of trans fats and refined carbohydrates has been associated with elevated TC (*β* = +0.45, *p* < 0.001) and LDL‐C (*β* = +0.39, *p* < 0.001) [20]. Urban populations demonstrate a hypertriglyceridemia prevalence of 34.1%, likely reflecting reduced adherence to traditional diets rich in fiber and unsaturated fats [7, 18].

#### 2.3.2. Obesity and Physical Inactivity

Current epidemiological data indicate that Saudi adults with a BMI > 30 comprise 24.7% of the population, while 15.9% of adolescents are classified as obese. However, emerging evidence suggests that the standard WHO cut‐off of 30.0 kg/m^2^ lacks diagnostic sensitivity for the Saudi population due to higher body fat percentages at lower BMI levels. Research specifically identifying Saudi‐specific thresholds suggests that a lower BMI cut‐off of 27.0 provides greater diagnostic accuracy for obesity‐related metabolic risk in this cohort. Regardless of the threshold used, obesity remains a primary driver of dyslipidemia through insulin resistance and altered lipoprotein metabolism. Compared to normal‐weight peers, obese adolescents face 2.8 times greater odds of developing dyslipidemia (95% CI: 2.34–3.34). Furthermore, physical inactivity—defined as less than 150 min of moderate‐intensity activity per week—affects 44.3% of adolescents and 55.5% of adults, further exacerbating lipid abnormalities by suppressing HDL‐C levels and elevating TG levels [8, 9].

#### 2.3.3. Diabetes Mellitus

The 25.2% prevalence of T2DM in Saudi Arabia substantially amplifies dyslipidemia risk, particularly in the setting of impaired glycemic control. This metabolic state promotes increased hepatic VLDL secretion and the inhibition of lipoprotein lipase, driving the characteristic lipid abnormalities seen in this population. People with diabetes show a 70% increased prevalence of combined dyslipidemia (high TG levels and low HDL‐C) when compared to nondiabetic individuals (OR = 1.7, 95% CI: 1.2–2.4) [14, 21].

#### 2.3.4. Smoking and Socioeconomic Factors

While cigarette smoking prevalence in Saudi Arabia is reported at 12.8%, the broader landscape of tobacco consumption—including the widespread use of shisha (waterpipe) and electronic cigarettes—increases total tobacco use to approximately 17.8%. Shisha use, in particular, remains a potent lipid disruptor; studies within the Saudi population indicate that shisha smokers often exhibit significantly higher atherogenic indexes and lower HDL‐C levels compared to nonsmokers, with some evidence suggesting the risk of low HDL‐C is 1.5 times greater in tobacco users (95% CI: 1.1–2.0). These findings reinforce the necessity of cessation programs that address all forms of tobacco consumption as a core component of cardiovascular risk management [19, 22].

### 2.4. Geographic Disparities

Geographic variation in dyslipidemia prevalence is evident across Saudi Arabia. The Northern and Eastern regions show the highest rates, which may be associated with higher levels of urbanization, sedentary behavior, and access to energy‐dense foods, while the Southern region reports substantially lower prevalence (14.1%), potentially reflecting continued adherence to traditional dietary patterns and more active lifestyles. Males show higher rates of hypertriglyceridemia (47.6% vs. 33.7%) and low HDL‐C (47.6% vs. 33.7%), yet females display elevated TC and LDL‐C levels, driven largely by hormonal shifts and body composition variations. Following menopause (typically after age 50), women experience a worsened lipid profile, with LDL‐C levels estimated to rise by approximately 0.2 mmol/L per decade during mid‐to‐late adulthood. However, this upward trajectory is not indefinite; consistent with global data, LDL‐C concentrations tend to plateau or even decline in the very elderly (typically after age 75–80), likely due to changes in nutritional status and biological frailty [7, 9, 23].

### 2.5. Pathophysiology of Lipid Disorders in Cardiovascular Mortality

Dyslipidemia is a well‐established modifiable risk factor for ASCVD and is strongly associated with increased cardiovascular morbidity and mortality. Rather than acting as a direct cause, lipid abnormalities contribute to atherogenesis through a series of interconnected biological processes that promote plaque development, progression, and instability. Understanding these mechanisms is essential for interpreting epidemiological associations and for designing preventive and therapeutic strategies tailored to population‐specific risk profiles, including those observed in Saudi Arabia [24].

### 2.6. Mechanisms of Lipid Abnormalities in Atherosclerosis

#### 2.6.1. Lipid Retention and Modification

Atherosclerosis develops through the retention of apolipoprotein B–containing lipoproteins within the arterial intima, followed by their structural modification and interaction with vascular cells. Cholesterol accumulation and chronic low‐grade inflammation represent central features of this process. LDL particles that penetrate the endothelial barrier become trapped in the subendothelial matrix, where they undergo oxidative, enzymatic, and compositional modifications. These modified LDL particles, including small dense, electronegative, and desialylated forms, exhibit increased affinity for arterial proteoglycans and enhanced susceptibility to further modification, thereby amplifying their atherogenic potential. Importantly, clinical manifestations of atherosclerosis do not arise from lipid oxidation itself but rather from plaque rupture, erosion, or progressive luminal narrowing, which occur after prolonged plaque development and remodeling.

#### 2.6.2. Inflammatory Cascade

Modified lipoproteins initiate and sustain vascular inflammation by activating endothelial cells, macrophages, and smooth muscle cells. This activation involves transcriptional pathways such as NF‐κB, leading to increased expression of adhesion molecules, cytokines, and chemokines. These signals promote monocyte recruitment, foam cell formation, and the development of fatty streaks, which represent early atherosclerotic lesions. Immune responses to modified lipoproteins, including the formation of circulating immune complexes, further contribute to inflammatory signaling and cellular dysfunction within the arterial wall. Collectively, these processes drive lipid accumulation, extracellular matrix remodeling, smooth muscle cell proliferation, and plaque progression rather than directly triggering acute cardiovascular events [25].

### 2.7. Low HDL‐C Levels and Mortality Among Saudis

Low levels of HDL‐C are highly prevalent in the Saudi population and represent the most frequently observed lipid abnormality [26]. Epidemiological studies consistently demonstrate an association between low HDL‐C levels and adverse cardiovascular outcomes, including higher mortality among patients with established CVD. In congestive heart failure patients from Saudi Arabia, low HDL‐cholesterol levels emerged as a significant independent predictor of substantial mortality risk, with an odds ratio of 1.29 (CI 1.04–1.59, *p* < 0.01) following adjustments for age, gender, and statin use. However, consistent with global observations, this association likely reflects a complex interplay where low HDL‐C serves as a marker of disease severity and reduced exercise tolerance, rather than functioning solely as an isolated causative factor [27].

Among Arab populations more broadly, reduced HDL‐C levels have also been associated with poorer functional recovery and increased recurrence rates following ischemic stroke. These findings support the role of HDL‐C as a marker of cardiovascular risk rather than a direct determinant of disease progression [28]. HDL particles participate in several protective biological processes, including reverse cholesterol transport, antioxidative activity, and modulation of vascular inflammation [28]. While these results highlight a strong prognostic link, current evidence suggests that low HDL‐C in this setting may largely function as a marker of overall frailty and physical inactivity associated with cerebrovascular disease, rather than acting purely as an isolated biological cause of recurrence.

However, pharmacological strategies aimed solely at increasing HDL‐C concentrations have not consistently translated into improved clinical outcomes. This discrepancy suggests that HDL functionality and overall lipid context, rather than HDL‐C levels alone, are more relevant for cardiovascular risk assessment [29].

### 2.8. Gender‐Specific Variations in Lipid Profiles and CVD Risk

Sex‐related differences in lipid profiles have important implications for cardiovascular risk assessment. Women generally exhibit higher TC and HDL‐C levels and lower TG concentrations than men, resulting in more favorable lipid ratios during premenopausal years [30]. In contrast, men more frequently display an atherogenic lipid pattern characterized by elevated TG and low HDL‐C, which is associated with earlier onset of CVD [31]. The principal sex‐related differences in lipid parameters reported in the literature are summarized in Table 2.

**TABLE 2:** Gender differences in lipid profiles.

Parameter	Males	Females	Clinical significance
Total cholesterol (TC)	Lower	Higher	Higher TC in females, but more favorable HDL ratio
HDL‐cholesterol	Lower	Higher	Protective effect more pronounced in premenopausal women
LDL‐cholesterol	Variable	Variable	Differences less consistent across studies
Triglycerides (TG)	Higher	Lower	Higher TG in males contributes to atherogenic profile
TC/HDL‐C ratio	Higher	Lower	Better predictor of CVD risk than individual parameters
High TG + low HDL‐C	Twice as frequent	Less frequent	Atherogenic dyslipidemia more common in males

Lipid profiles change across the life course in both sexes and are influenced by hormonal status, aging, and cumulative exposure to metabolic risk factors [32]. During puberty and adulthood, sex‐specific differences in cholesterol fractions emerge and evolve. After menopause, women experience unfavorable shifts in lipid profiles, including rising LDL‐C levels, which coincide with an increased incidence of CVD. In the Saudi population, gender differences in cardiovascular risk reflect both biological and behavioral factors. While physical inactivity and unhealthy dietary patterns are common in both sexes, obesity is more prevalent among women, whereas smoking and dyslipidemia are more common among men. These patterns contribute to differing risk trajectories and underscore the importance of sex‐specific risk stratification. Age‐ and sex‐specific distributions of major cardiovascular risk factors in the Saudi population are presented in Table 3 [32].

**TABLE 3:** Age and gender‐specific cardiovascular risk factors in the Saudi population.

Risk factor	Males (%)	Females (%)	Age group most affected
Smoking	28.0	2.7	18–29 years (males)
Obesity	37.9	62.9	≥ 60 years (both sexes)
Diabetes	38.7	50.0	≥ 60 years (both sexes)
Hypertension	15.0	19.5	50–59 years (females)
Hypercholesterolemia	50.7	53.4	50–59 years (both sexes)
High 10‐year ASCVD risk	32.0	7.6	Increases with age
Lifetime ASCVD risk	67.0	51.0	Young adults

Assessment of cardiovascular risk should therefore incorporate age, sex, menopausal status, and composite lipid measures such as cholesterol ratios, rather than relying on isolated lipid parameters. Tailored preventive strategies—such as weight management interventions for women and smoking cessation combined with lipid optimization for men—are essential for addressing gender‐specific cardiovascular risk in Saudi Arabia.

## 3. Clinical Evidence Linking Lipid Disorders to Cardiovascular Mortality in Saudi Arabia

In Saudi Arabia CVD makes up over 45% of total mortality cases, and dyslipidemia stands out as an important preventable risk element [26, 33]. Research from local epidemiological studies indicates disturbingly elevated occurrences of lipoprotein imbalances characterized by low HDL‐C levels and high TG numbers which show independent links to coronary artery plaque development and increased mortality [20, 34, 35]. This section combines findings from Saudi Arabia about the predictive importance of HDL‐C and TG levels for CVD outcomes to offer population‐specific risk assessment and management strategies. In Saudi Arabia, low HDL‐C stands as the most common lipid abnormality impacting 46.3% of young adults and 82.9% of congestive heart failure patients [20, 26]. A 12‐year retrospective cohort study of 485 elderly patients (≥ 60 years) in Riyadh’s primary care settings demonstrated that individuals in the lowest HDL‐C quartile (< 1.1 mmol/L) faced a twofold increased risk of all‐cause mortality (adjusted HR: The study revealed that those with HDL‐C values in the lowest quartile had double the risk of dying from any cause (adjusted HR: 2.02; 95% CI: 1.21–3.38) and triple the risk of developing ischemic heart disease (adjusted HR: 3.2; 95% CI: 1.6–6.2) [36]. The coronary computed tomography (CT) study of 2421 Saudi patients without prior CAD revealed that males experienced an 18% reduction in odds for soft or calcified plaque formation with each 0.36 mmol/L increase in HDL‐C levels (OR: 0.82; 95% CI: 0.71–0.95) [34].

Low HDL‐C levels lead to significant mortality impacts for HF populations. A single‐center study of 392 Saudi HF patients revealed that 82.9% had HDL‐C levels below the recommended threshold, contributing to a 29% increase in mortality risk (OR: 1.29; 95% CI: 1.04–1.59) upon controlling for statin treatment and other medical conditions. Notably, this risk persisted even though 70% of participants were on statin therapy, highlighting the limitation of relying solely on LDL‐C reduction. However, clinically, this low HDL‐C likely also functions as a marker of disease severity and physiological frailty in advanced heart failure [26, 37] (see Tables 4, 5, and 6).

**TABLE 4:** Key Saudi studies on HDL‐C and cardiovascular outcomes.

Study population	Design	Key findings	Reference
Elderly (≥ 60 years)	Retrospective cohort	HDL‐C < 1.1 mmol/L linked to 2 × higher mortality and 3 × higher IHD risk	37
Heart failure patients	Retrospective	82.9% had low HDL‐C; 29% increased mortality risk independent of statins	27
General adults	Cross‐sectional	46.3% prevalence of low HDL‐C, highest among males and sedentary individuals	20

**TABLE 5:** Triglyceride‐associated plaque burden in Saudi studies.

Study design	Population	TG threshold	Plaque type	Risk increase	Reference
Retrospective cross‐sectional	2421 adults	≥ 1.64 mmol/L	Soft plaques (males)	OR: 2.1 (1.4–3.2)	35
Retrospective cohort	1541 adults	TG/HDL > 3.5	Major cardiac events	HR: 1.68 (1.22–2.31)	36
Cross‐sectional	2732 adults	≥ 2.26 mmol/L	Atherogenic dyslipidemia	27.8% prevalence	40

**TABLE 6:** Various types of lipid‐lowering medications available in Saudi Arabia.

Therapy	Mechanism	LDL‐C reduction	HDL‐C effect	Triglyceride effect	Considerations in Saudi Arabia
Statins	HMG‐CoA reductase inhibition	37%–61%	+5%–10%	10%–20%	First‐line; limited effect on HDL‐C/TG
Ezetimibe	Cholesterol absorption blockade	15%–25%	Neutral	Neutral	Add‐on therapy; improves LDL‐C control
PCSK9 inhibitors	LDL receptor upregulation	50%–60%	Neutral	Neutral	Effective for FH; cost/access challenges
Bempedoic acid	ACL inhibition (liver‐specific)	18%–24% (mono), up to 40% (combo)	Neutral	Neutral	Suitable for statin intolerance; emerging use
Pemafibrate (selective PPAR‐α modulator)	Triglyceride lowering, HDL‐C increase	5%–20%	+10%–15%	42%–48%	Promising for hypertriglyceridemia; safety profile favorable
Niacin	Inhibits VLDL synthesis	10%–20%	+15%–35%	20%–30%	Limited by side effects; less used currently

Coronary plaque morphology shows strong associations with elevated levels of TG (> 1.64 mmol/L) which occur in 35% of Saudi adults [34, 38]. In a CT angiography study of 2421 patients, high TG levels independently predicted soft plaque formation in both sexes, with males exhibiting a stronger association (OR: 1.24 per 1.09 mmol/L increase; 95% CI: 1.08–1.43). Gender disparities were notable: Men with TG levels ≥ 1.64 mmol/L faced 2.1 times higher risk for mixed calcified/soft plaques compared to females who showed a 1.7 times increase [34]. The atherogenic potential of TG is amplified when combined with low HDL‐C. A 2023 study of 1541 Saudi patients demonstrated that a TG/HDL‐C ratio > 3.5 increased the risk of major adverse cardiac events by 68% (HR: 1.68; 95% CI: 1.22–2.31) [36]. This ratio outperformed LDL‐C in predicting long‐term mortality, particularly in patients with metabolic syndrome components such as obesity (prevalence: 31%) and diabetes (prevalence: 39.6%) [39].

The lipid profile of the Saudi population stands out due to its low HDL‐C levels along with growing TG concentrations. A mix of genetic predispositions together with inactive living patterns and consumption of foods high in refined carbohydrates leads to this condition [34]. High rates of vitamin D deficiency between 50% and 90% in Saudis create additional obstacles for HDL‐C function by blocking reverse cholesterol transport mechanisms and anti‐inflammatory pathways [40]. High levels of TG‐rich lipoproteins promote endothelial dysfunction through the increased expression of apolipoprotein C‐III which plays a major role in plaque instability [41]. The findings highlight the importance of addressing residual lipid risk, while standard statin therapy remains the cornerstone, intensive lifestyle interventions and omega‐3 fatty acid supplementation (such as icosapent ethyl) offer guideline‐compliant avenues to improve the overall lipid profile in high‐risk patients. Primary care providers should routinely measure TG/HDL‐C ratios and use fibrates or icosapent ethyl to treat patients with ratios greater than 3.5 [42]. It is critical to follow gender‐specific guidelines because males need aggressive TG level management while females benefit from HDL‐C optimization due to their unique plaque formation risks [43].

## 4. Current Management Strategies for Lipid Disorders in Saudi Arabia

Elevated LDL‐C, low HDL‐C, and high TG levels together create dyslipidemia, which stands as the top modifiable CVD risk factor worldwide. Saudi Arabia faces a high rate of lipid abnormalities, together with growing obesity and diabetes rates and inactive lifestyles [44, 45]. Healthcare professionals need to understand standard treatment options for lipid disorders and their limitations, while also developing culturally adapted lifestyle interventions—such as dietary modifications and physical activity plans respectful of local norms—to manage this condition effectively in this population. While statins remain the cornerstone of dyslipidemia management due to their proven efficacy in LDL‐C reduction, current evidence highlights the need to address residual risk in specific populations. Statins represent the primary treatment for dyslipidemia because they lower LDL‐C by blocking HMG‐CoA reductase, which boosts LDL receptor activity and aids in cholesterol removal. Statins, including atorvastatin and rosuvastatin, can achieve LDL‐C reductions between 37% and 61% which leads to substantial declines in cardiovascular event rates according to major studies [46, 47]. Statins effectively reduce LDL‐C levels but show limited effectiveness in increasing HDL‐C and decreasing TG, which are key elements of atherogenic dyslipidemia common among Saudis. The range of therapies available for lipid management has grown through the inclusion of adjunctive treatments. The cholesterol absorption inhibitor Ezetimibe delivers an additional LDL‐C reduction of 15%–25% when used with statins to improve lipid control for patients who fail to reach lipid goals or cannot tolerate statins [48]. Alirocumab and evolocumab are two monoclonal antibodies known as PCSK9 inhibitors, which serve as potent LDL‐C reduction agents with efficacy between 50% and 60%. Patients with familial hypercholesterolemia and individuals facing very high cardiovascular risk benefit significantly from these agents [49]. Bempedoic acid functions as an inhibitor of ATP‐citrate lyase in the liver to achieve LDL‐C reduction between 18% and 24% alone and up to 40% when used with ezetimibe while maintaining safety for statin‐intolerant patients [50].

LDL‐C reduction continues as the main goal even though Saudi Arabia faces difficult problems with low HDL‐C management and high TG levels. According to epidemiological studies, 83% of adult individuals experience low HDL‐C, and hypertriglyceridemia exists in 44% of them, demonstrating a connection to widespread atherogenic dyslipidemia in relation to the high obesity rate of 24.7% and diabetes rate of 25.2% in the region [51]. The combination of diets containing refined carbohydrates and saturated fats with prevalent physical inactivity and sedentary lifestyles worsens lipid abnormalities. There are few medications available to treat low HDL‐C and hypertriglyceridemia. Selective PPAR‐α modulators like pemafibrate demonstrate significant TG reduction of 42%–48% and slight increases in HDL‐C while maintaining better safety profiles than traditional fibrates [52]. Niacin has been traditionally used to increase HDL‐C and decrease TG, but its use is restricted because it causes side effects like flushing and negative effects on glycemic control. Statins provide only minor enhancements to these biomarkers which emphasizes the importance of complete lifestyle modifications and new treatment methods. Patients with complex lipid profiles or who experience statin intolerance may find effective treatment alternatives in new pharmacotherapies such as bempedoic acid and pemafibrate. The combined treatment of bempedoic acid and ezetimibe achieves significant LDL‐C reduction while producing minimal muscle‐related adverse effects which is crucial for patients with statin intolerance in Saudi Arabia [53]. Despite facing limitations from high costs and restricted access, PCSK9 inhibitors remain essential treatments for familial hypercholesterolemia and extremely high‐risk patients according to Sabatine et al. [49]. The primary approach to dyslipidemia management involves lifestyle modification which takes into account Saudi Arabia’s specific cultural and environmental contexts. The Mediterranean‐style diet, which emphasizes olive oil, whole grains, fruits, and vegetables while minimizing processed foods and sugary drinks, matches traditional eating habits and shows positive effects on lipid profiles. Culturally appropriate physical activities, such as community walking programs on traditional Samma paths, help reduce sedentary behavior. For patients with T2DM, addressing vitamin D deficiency through supplementation may serve as a valuable adjunct, as emerging evidence suggests it can improve HDL‐C levels and overall metabolic health within this specific cohort.

## 5. Public Health Implications

### 5.1. The Burden of Lipid Disorders on Saudi Healthcare Systems

CVDs represent a substantial public health challenge in Saudi Arabia, with lipid disorders constituting a major associated risk factor within a broader cardiometabolic context [33]. The increasing prevalence of dyslipidemia contributes to rising healthcare utilization through long‐term disease management, recurrent hospital admissions, and treatment of cardiovascular complications. In 2016, the estimated economic impact of CVD in Saudi Arabia reached $3.5 billion, reflecting both direct healthcare expenditures and indirect productivity losses [54, 55].

**FIGURE 1:**
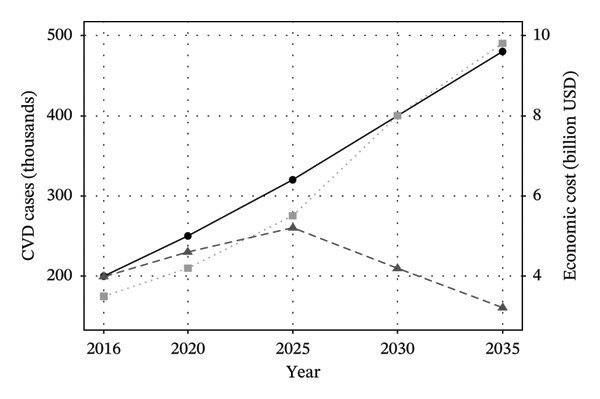
Projected cardiovascular disease (CVD) burden and economic impact in Saudi Arabia under current trends and WHO intervention scenarios (2016–2035). Note: Solid lines represent projected numbers of CVD cases assuming continuation of current trends, while dashed lines illustrate modelled estimates assuming implementation of World Health Organization (WHO)–recommended population‐level risk factor reduction strategies. The dotted line represents projected economic costs (right *y*‐axis). Estimates are based on published national data and modelling assumptions and do not represent observed longitudinal outcomes. Line styles and grayscale formatting were selected to ensure readability in print and accessibility for color‐blind readers.

As illustrated in Figure 1, projections based on current epidemiological trends suggest a continued rise in CVD burden over time, accompanied by increasing healthcare and economic pressures if no effective preventive strategies are implemented. These modeled projections indicate a substantial growth in the number of individuals affected by CVD, emphasizing the long‐term implications for healthcare capacity and resource allocation. In addition to financial considerations, CVD is a leading contributor to disability and reduced quality of life, as reflected by high rates of disability‐adjusted life years lost in the Saudi population.

From a public health perspective, the trends shown in Figure 1 highlight the importance of early intervention and comprehensive risk factor modification. Addressing lipid abnormalities through population‐based screening, lifestyle interventions, and optimized clinical management represents a critical opportunity to attenuate future cardiovascular burden. Integrating dyslipidemia prevention and control into broader national cardiovascular health strategies may help limit the projected trajectory of disease burden while improving long‐term population health outcomes.

### 5.2. Importance of Early Screening and Prevention Programs

Early identification of lipid disorders represents a key component of CVD prevention and has the potential to reduce long‐term disease burden and healthcare utilization [8, 56]. Available data indicate that a substantial proportion of individuals with dyslipidemia remain undiagnosed or unaware of their condition, underscoring gaps in current detection strategies. These findings highlight the need for structured and systematic screening approaches to identify at‐risk individuals before the onset of clinically apparent CVD [8, 57].

Current Saudi Clinical Preventive Guidelines recommend fasting lipid profile screening in adults, with particular emphasis on individuals with diabetes, hypertension, or a family history of premature CVD [58]. Early screening may also facilitate the identification of inherited lipid disorders, such as familial hypercholesterolemia, which remains underdiagnosed in Saudi Arabia despite affecting a sizable segment of the population [59, 60]. From a public health perspective, integrating lipid screening into routine primary care provides an opportunity for timely risk stratification and initiation of preventive interventions.

### 5.3. Current Public Health Initiatives and Their Impact

Saudi Arabia has implemented multiple public health initiatives aimed at reducing cardiovascular risk factors through lifestyle modification and population‐level interventions [61, 62]. Programs such as the Obesity Control Programme and the Diet and Physical Activity Program have focused on promoting healthier dietary patterns and increasing physical activity across different age groups [63]. More targeted initiatives, including the RASHAKA Program, emphasize weight management and behavioral change among adults [62].

Evidence from localized and pilot‐level studies suggests that intensive lifestyle interventions delivered in primary care settings can achieve clinically meaningful improvements in weight and metabolic risk factors [64]. In addition, culturally adapted programs, such as the Saudi‐modified Tawazon Diabetes Prevention Program, demonstrate the feasibility and acceptability of context‐specific approaches that incorporate religious and cultural considerations [65].

However, many national initiatives are ongoing, and comprehensive long‐term outcome data evaluating their sustained impact on lipid profiles and cardiovascular events remain limited. Continued monitoring, standardized evaluation frameworks, and integration of outcome metrics are needed to assess effectiveness and inform the refinement and scaling of these programs at the national level. An overview of major national and community‐based public health initiatives targeting cardiovascular risk factors in Saudi Arabia is presented in Table 7.

**TABLE 7:** Saudi public health initiatives targeting cardiovascular risk factors.

Program/Initiative	Year started	Target population	Key components	Status
Obesity Control Programme	2013	All age groups	Healthy lifestyle promotion	Ongoing
Diet and Physical Activity Program	2006	General population	Dietary counseling, physical activity	Ongoing
RASHAKA Program	2017	Adults (≥ 18 years)	Weight management, lifestyle modification	Ongoing
Tawazon Diabetes Prevention Program	2017	Prediabetic adults (18–65 years)	Intensive lifestyle intervention	Pilot completed
Saudi Clinical Preventive Guidelines	2023	Adults (Screening starting at age 20; Risk calculation at 40–75)	Lipid screening recommendations	Active
National Screening Programs	2010	Adults (≥ 30 years for NCD screening)	Diabetes and hypertension screening	Active
Sports Boulevard Project	2019	Riyadh residents	Community exercise facilities	Ongoing
Saudi Vision 2030 Healthcare Transformation	2016	National population	Digital health, prevention focus	Active
Johnson and Johnson AFib Awareness Campaign	2022	Adults > 40 years	Atrial fibrillation education	Active
WHO HEARTS Package Implementation	Planned	Primary care patients	CVD risk management protocols	In development

**FIGURE 2:**
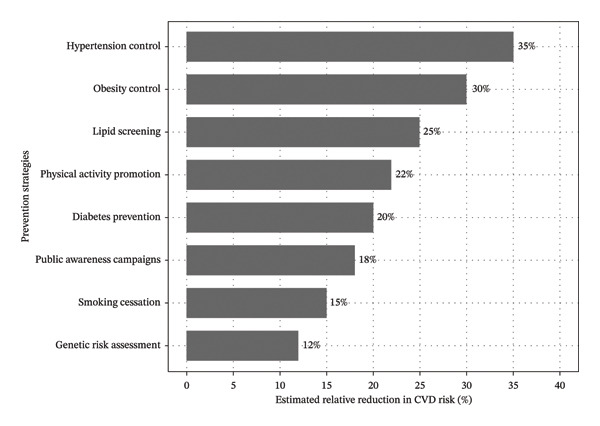
Modeled potential impact of selected cardiovascular disease prevention strategies in Saudi Arabia. Note: Estimated relative risk reductions represent synthesized evidence from international studies and regional reports applied to the Saudi context for illustrative purposes. Values assume optimal implementation and population adherence and reflect hypothetical effects rather than measured outcomes from national programs.

The range of prevention strategies currently implemented or under development and their potential population‐level effects are summarized in Figure 2. This figure presents modeled estimates of relative cardiovascular risk reduction based on synthesized evidence from international studies and regional reports and is intended to illustrate the potential impact of comprehensive prevention approaches rather than to represent observed outcomes from Saudi national programs. Furthermore, national screening protocols now emphasize that cardiovascular risk assessment should begin for all asymptomatic adults starting at age 40, though screening for individual risk factors like BMI and blood pressure often commences at age 20.

### 5.4. Strategies to Address Modifiable Risk Factors

Saudi Arabia needs comprehensive multilevel interventions due to the widespread presence of modifiable risk factors [21]. Saudi nationals show higher obesity rates of 52.6% and abdominal obesity rates of 65.5% than expatriates, along with troubling physical inactivity levels that affect 44.3% of adolescents and 55.5% of adults. Success‐oriented approaches need to consider the Saudi population’s unique genetic and environmental interactions [27, 66]. The dyslipidemia epidemic has been significantly driven by the shift to Western dietary patterns which emphasize high intake of refined carbohydrates and trans fats. Promising approaches to health improvement combine traditional dietary pattern interventions with accessible physical activity programs like the Sports Boulevard Project in Riyadh [67].

Saudi Vision 2030’s inclusion of digital health solutions enables widespread population intervention opportunities. Healthcare accessibility has improved through platforms like Sehhaty and Mawid which also enable large‐scale prevention programs. The introduction of the WHO HEARTS package aims to systematize cardiovascular risk management through primary care interventions which serve as preventive measures [68].

## 6. Future Directions

### 6.1. Gaps in Research on Lipid Disorders and Cardiovascular Mortality

Despite the substantial burden of lipid disorders in Saudi Arabia, important gaps remain in the current evidence base. A major limitation is the scarcity of longitudinal epidemiological studies capable of assessing the long‐term relationship between lipid abnormalities and cardiovascular mortality. Most available data are derived from cross‐sectional or retrospective analyses, which limit the ability to evaluate disease progression, temporal associations, and long‐term outcomes [8, 56, 69].

In addition, although several genetic polymorphisms, such as those involving PCSK9, CETP, and CDKN2B‐AS1, have been associated with increased cardiovascular risk in Saudi populations, comprehensive genome‐wide association studies remain limited. The lack of Saudi‐specific genetic reference datasets restricts the integration of genetic risk into routine cardiovascular assessment. Fragmentation of healthcare data across institutions further complicates population‐level analyses and hinders the development of unified national datasets [8].

### 6.2. Need for Large‐Scale Epidemiological Studies and Clinical Trials

The establishment of a national CVD registry represents a critical step toward addressing current knowledge gaps. Such a registry would enable standardized data collection, longitudinal follow‐up, and evaluation of treatment outcomes across diverse patient groups. Local professional societies have emphasized the importance of registries, particularly given the high prevalence of diabetes and metabolic comorbidities among patients with established CVD [70].

There is also a need for randomized controlled trials designed within the Saudi context to assess the effectiveness of prevention and treatment strategies. While community‐based and lifestyle interventions have demonstrated benefits internationally, their long‐term effectiveness and scalability within Saudi Arabia require further evaluation [71]. Pilot programs, including culturally adapted lifestyle interventions, suggest feasibility but remain limited by short follow‐up durations and modest sample sizes. Community‐based interventions have proven effective in reducing cardiovascular risks on a global scale but require thorough examination to determine their efficacy in achieving predefined, target‐based outcomes within the Saudi Arabian context. Specifically, these interventions must be evaluated against national benchmarks—such as the reductions in physical inactivity and hypertension prevalence outlined in the Saudi Vision 2030 Health Sector Transformation Program. The positive results from pilot studies, including Tawazon, confirm that Saudi Arabia possesses the capacity to conduct high‐quality intervention research centered on reaching these specific clinical and behavioral targets.

Future clinical trials should prioritize lipid patterns common in Saudi populations, particularly low HDL‐C levels and hypertriglyceridemia, which are frequently observed in high‐risk groups such as patients with heart failure. Investigating combination therapies and alternative lipid targets may help address residual cardiovascular risk not fully captured by LDL‐C–focused strategies [8, 26, 56, 72].

### 6.3. Recommendations for Personalized Medicine Approaches

National genomic initiatives provide an important foundation for advancing personalized cardiovascular care in Saudi Arabia. Large‐scale sequencing efforts offer opportunities to identify population‐specific genetic variants associated with lipid disorders and cardiovascular risk [60]. Integrating genetic screening with conventional risk assessment tools may improve early identification of high‐risk individuals, particularly in a population with high consanguinity rates [69, 73].

Current international risk calculators may not accurately reflect the Saudi cardiovascular risk profile, highlighting the need for locally derived prediction models [8, 60]. Pharmacogenomic approaches may also support more individualized lipid‐lowering therapy, especially in patients who show suboptimal responses to standard treatments. The integration of genomic data, biomarkers, and clinical characteristics into routine care, supported by specialized precision medicine clinics, represents a promising but still evolving direction for CVD prevention and management in Saudi Arabia.

## 7. Conclusion

This review identifies lipid disorders as a major public health concern in Saudi Arabia and highlights their strong association with cardiovascular morbidity and mortality. Dyslipidemia is highly prevalent, with a characteristic pattern of low HDL‐C and elevated TG levels commonly observed among individuals with CVD. This profile suggests substantial residual cardiovascular risk that is not fully addressed by treatment strategies focused primarily on LDL‐C. Cardiovascular risk in the Saudi population reflects a complex interaction of metabolic, lifestyle, and genetic factors. High rates of obesity, diabetes, and physical inactivity contribute to adverse lipid profiles, while population‐specific genetic characteristics may further influence susceptibility to atherosclerotic disease. Although current lipid‐lowering therapies are effective for LDL‐C reduction, they are less successful in addressing low HDL‐C levels and hypertriglyceridemia, underscoring the need for broader risk assessment and management approaches. Improving cardiovascular outcomes will require early detection through population‐based screening, implementation of culturally adapted lifestyle interventions, and integration of personalized risk assessment strategies. Future priorities include large‐scale epidemiological studies, national cardiovascular registries, and the development of Saudi‐specific risk prediction tools to guide prevention and management efforts.

## Author Contributions

Aseel Awad Alsaidan: sole responsibility for study conception, design, data collection, analysis, interpretation of results, and manuscript preparation.

## Funding

No funding was received for this manuscript.

## Disclosure

The author confirms that this is an original manuscript and has not been previously published or submitted to another journal.

## Conflicts of Interest

The author declares no conflicts of interest.

## Data Availability

The data used to support the findings of this study are included within the article.
